# From predator to protector: *Myxococcus fulvus* WCH05 emerges as a potent biocontrol agent for fire blight

**DOI:** 10.3389/fmicb.2024.1378288

**Published:** 2024-04-08

**Authors:** Jian Han, Zhiming Dong, Wenbo Ji, Wen Lv, Ming Luo, Benzhong Fu

**Affiliations:** ^1^Department of Plant Pathology, College of Agronomy, Xinjiang Agriculture University/Key Laboratory of the Pest Monitoring and Safety Control of Crops and Forests of Xinjiang Uygur Autonomous Region, Urumqi, China; ^2^Key Laboratory of Prevention and Control of Invasive Alien Species in Agriculture and Forestry of the North-western Desert Oasis (Co-construction by Ministry and Province), Ministry of Agriculture and Rural Affairs, Urumqi, China

**Keywords:** *Myxococcus fulvus* WCH05, *Erwinia amylovora*, predation, biocontrol, extracellular proteins, fire blight

## Abstract

Fire blight, caused by the Gram-negative bacterium *Erwinia amylovora*, poses a substantial threat to pome fruit production worldwide. Despite existing control strategies, a pressing need remains for sustainable and environmentally friendly fire blight management. Myxobacteria, renowned for their predatory behavior and potent enzymes, emerge as a groundbreaking biocontrol approach with significant potential. Here, we report the biocontrol potential of a novel *Myxococcus fulvus* WCH05, against *E. amylovora*. Using various *in vitro* and planta assays, we demonstrated the multifaceted biocontrol abilities of strain WCH05. In plate predation assays, strain WCH05 exhibited not only strong predation against *E. amylovora* but also broad-spectrum activities against other plant pathogenic bacteria. Pre-treatment with strain WCH05 significantly decreased pear blossom blight incidence in detached inflorescence assays, achieving a controlled efficacy of 76.02% that rivaled the antibiotic streptomycin (79.79%). In greenhouse trials, strain WCH05 effectively reduced the wilting rate and disease index in young pear seedlings, exhibiting both protective (73.68%) and curative (68.66%) control. Further investigation revealed that the biocontrol activity of strain WCH05 relies on both direct contact and extracellular enzyme secretion. While cell extracts lacked inhibitory activity, ammonium sulfate-precipitated secreted proteins displayed potent lytic activity against *E. amylovora*. Substrate spectrum analysis identified peptidases, lipases, and glycosidases among the secreted enzymes, suggesting their potential roles in pathogen degradation and biocontrol efficacy. This study presents the first evidence of *Myxococcus fulvus* WCH05 as a biocontrol agent against fire blight. Its potent predatory abilities and enzymatic arsenal highlight its potential for sustainable disease management in pome fruit production. Future research will focus on identifying and characterizing specific lytic enzymes and optimizing strain WCH05 application strategies for field efficacy.

## 1 Introduction

Fire blight, caused by the bacterium *Erwinia amylovora*, stands as one of the most devastating bacterial diseases affecting pome fruit trees within the *Rosacea*e family (Malnoy et al., 2012). This disease poses a significant threat to pear, apple, hawthorn, and quince trees, with a particularly rapid spread observed in pear fields (Momol et al., 2000). The bacterium can infect the blossoms of fruit trees and serve as a source of infection for leaves, young shoots, and immature fruit. Additionally, wounded branches represent a major way for bacterial invasion. Once the pathogen infiltrates the plant, it establishes lifelong colonization, leading to dissemination throughout the entire plant, rapid proliferation, and challenging control and eradication (Huang et al., 2022). In 2016, fire blight was first diagnosed in Yili region of Xinjiang Autonomous Region, located in northwestern China. Subsequently, in 2017, a widespread outbreak occurred in the Korla region of Xinjiang, resulting in an overall production reduction of approximately 30 to 50%. The disease later spread to most pear-producing areas in Xinjiang and some regions in Gansu Province, causing severe damage (Sun et al., 2023).

Management measures for the control of fire blight disease include quarantine, pruning, and removal of diseased plants, chemical control, biological control, and breeding of resistant varieties (Johnson and Stockwell, 1998). However, there is currently a lack of fire-blight-resistant fruit tree varieties in production. Removing diseased branches can effectively prevent the spread of fire blight, but it severely affects fruit yield. The widespread use of chemical pesticides has led to increasing problems of pathogen resistance, environmental pollution, and pesticide residues (Russo et al., 2008; Tancos et al., 2016).

Biocontrol of the fire blight is a promising alternative way in orchard disease management. Currently, there are some commercial biocontrol agents available in the market (Mikiciński et al., 2019). These beneficial strains used for disease management mainly rely on their antibiotic substances (Temple et al., 2004), nutrient and niche competition, and induced host resistance (Van Wees et al., 1997; Pieterse et al., 2014).

Fire blight is a newly emerging plant disease in China, and research on its biocontrol has not accomplished much. Though there are few reports on endophytic bacteria *Klebsiella* sp. TN50, *Paenibacillus* sp. HN89 and *Pseudomonas* sp. SN37, *Bacillus velezensis* JE4 and FX1 with the potential for the disease control (Xu et al., 2021; Huang et al., 2022; Lv et al., 2022).

Myxobacteria are a group of higher-order prokaryotes with multicellular group behavior and complex life history. They can obtain nutrients by using living microbial cells or other macromolecules as food (Munoz-Dorado et al., 2016), and can also produce a variety of enzymes and secondary metabolites with antibacterial activity (Thiery and Kaimer, 2020). In addition, myxobacteria can form stress-resistant fruiting bodies and myxospores, making them have strong environmental adaptability and colonization ability (Dawid, 2000). These characteristics of myxobacteria make them a new type of biocontrol microbial resource. The current research and application of myxobacteria in the biological control of fire blight have not been reported.

In recent years, some greenhouse and field experiments have shown that the application of myxobacteria can significantly reduce the damage of seedling wilt of forest trees (Dahm et al., 2015), cucumber wilt (Li et al., 2017), pepper anthracnose (Yun, 2014), and rice blast (Shen et al., 2023). However, there are only a few research on plant bacterial diseases. Such as on carrot soft rot caused by *Pectobacterium carotovorum* subsp. *carotovorum* (Li et al., 2018) and tomato bacterial wilt caused by *Ralstonia solanacearum* (Dong et al., 2021). However, these studies are currently limited to soil-borne bacterial diseases, and there are no reports on bacterial diseases that spread mainly on the aboveground parts of plants, such as fire blight. This study aimed to evaluate the biocontrol potential of *Myxococcus fulvus* WCH05 (Dong et al., 2024) obtained in previous research with strong predatory activity against *E. amylovora* and to preliminarily explore its biocontrol mechanism.

## 2 Materials and methods

### 2.1 Bacteria and culture conditions

The phytopathogen bacterial strains employed in this study included six bacterial pathogens. *Dickeya fangzhongdai* (Df; GMCC 1.15464) and *Pectobacterium carotovorum* subsp. *carotovorum* (Pcc; Eu 678364) were kindly provided by Associate Professor Li Zhoukun from Nanjing Agricultural University. *Acidovorax citrulli* (Ac; FC440) (Chen et al., 2023) was generously donated by Professor Liu Jun from Xinjiang Agricultural University. *P. syringae* pv. *syringae* (Pss; ATCC 19310) and *Clavibacter michiganensis* subsp. *michiganensis* (Cmm; SHBCC D10416) were gifts from Researcher Zhang Xianglin at the Urumqi Customs. *Erwinia amylovora* (Ea; E.a001) (Lü et al., 2023) was isolated, identified, and preserved by our laboratory from pear tree branches infected with fire blight in a pear orchard in Korla City, Xinjiang, and has been determined to be a highly virulent strain. All strains were routinely cultured on Luria-Bertani (LB) broth (5 g/L yeast extract, 10 g/L tryptone, 10 g/L NaCl, pH 7.0) at 30°C. The strain WCH05 was isolated from the unvegetated desert soil (soil type: brown calcareous soil) in Manas County, Xinjiang, China (N: 44°53′48.06″, E: 89°01′54.77″). Sampling was conducted in August 2020. It was identified as belonging to the *Myxococcus fulvus* in our previous work (Dong et al., 2024). Myxobacteria strains were maintained on VY/2 (5 g/L yeast, 1 g/L CaCl_2_·2H_2_O, 15 g/L agar, pH 7.2) (Zhang et al., 2013) or Casitone-Tris (CTT) (10 g/L casitone, 1.97 g/L MgSO_4_, 1.21 g/L Tris–HCl, 0.21 g/L K_2_HPO_4_, 15 g/L agar, pH 7.6) (Nair et al., 2019) medium at 30°C. TPM (10 mM/L Tris–HCl, 1 mM/L KH_2_PO_4_, 8 mM/L MgSO_4_, pH 7.6), LBS (7 g/L soluble starch, 5 g/L yeast extract, 1 g/L casitone, 1 g/L MgSO_4_·7H_2_O, pH 7.0).

### 2.2 Predatory activity against plant pathogens

The predatory capabilities of strain WCH05 were evaluated against above mentioned six bacterial pathogens. Target bacteria suspensions (50 μL, OD_600_ = 1.0) were spread onto TPM plates to form prey lawns, followed by drying. A 3 μL droplet of strain WCH05 suspension was then placed 2 mm from the prey’s edge, initiating the encounter. Plates were incubated at 30°C for 5 days. Predatory expansion of the strain WCH05 swarm was visually monitored to assess its success in consuming and conquering the prey territory (Berleman et al., 2006).

To quantify the predatory performance, the bacterial lawn was removed using a sterile inoculating loop and suspended in 1 mL of sterile water. The dilution plating method was employed to enumerate the colonies of the six pathogenic bacteria, and the remaining viable cell count was calculated to assess the predatory capacity. It is noteworthy that strain WCH05 is unable to proliferate on LB medium. Images were captured in each case to visually document the predatory process. An SM7 Motic microscope was used for the image. Furthermore, co-cultures of strain WCH05 and *E. amylovora* on TPM plates were visualized using scanning electron microscopy (SEM, SUPRA55 VP, Zeiss).

### 2.3 Biological assay of strain WCH05 against fire blight

#### 2.3.1 Preparation of the inoculum of myxobacteria and pathogenic bacteria

The strain WCH05 was activated and inoculated into 3 mL of LBS liquid medium at 30°C and 160 rpm for 2 days. It was then inoculated into 200 mL of VY/2 medium at 30°C and 160 rpm for 3 days. The bacterial pellet at the bottom of the bottle was fully dispersed with a pipette to obtain the myxobacteria inoculum.

The *E. amylovora* was activated by picking a single colony into LB liquid medium. It was then cultured in a shaker at 28°C and 160 rpm for 24 h until the OD_600_ of the culture reached 1.0. It was then diluted with sterile water to a concentration of 10^7^ cfu·mL^−1^ as the inoculum.

#### 2.3.2 Assay on pear inflorescences

Pear (*Pyrus sinkiangensis*) inflorescences were collected from a pear orchard (Xinjiang Korla City) and inserted into 0.05% NaCl solution to keep them moist and prevent decay. The strain WCH05 bacterial suspension was sprayed onto the pear inflorescences using a hand-held sprayer. After incubating for 24 h in a chamber at 28°C and 70% relative humidity, *E. amylovora* bacterial suspension was sprayed onto the inflorescences. Disease development was observed and recorded at 3- and 5-days post-inoculation. Flower rot rate and efficacy were calculated. 4,000-fold diluted streptomycin (Huabei Pharmaceutical Factory, with an active ingredient content of 72%), strain WCH05, and sterile water were used as positive and negative control treatments, respectively.


$$\mathrm{Flower}\;\mathrm{rot}\;\mathrm{rate}\;\left(\%\right)=\left(\begin{array}{l} \mathrm{number of diseased flowers}/\\ \mathrm{total number of flowers} \end{array}\right)\times 100\%\text{.}$$

$$\begin{array}{c} \mathrm{Efficacy}\;\left(\%\right)=\left(\begin{array}{l} \mathrm{negative control flower}\;\mathrm{rot}\;\mathrm{rate}-\\ \mathrm{treatment flower}\;\mathrm{rot}\;\mathrm{rate} \end{array}\right)/\\ \mathrm{negative control flower}\;\mathrm{rot}\;\mathrm{rate}\times 100\%\text{.} \end{array}$$

#### 2.3.3 Assay on potted pear seedlings

##### 2.3.3.1 Protective assay

The experiment was conducted in a greenhouse using 2-year-old potted pear seedlings (*P. betulifolia*) as the test material. The strain WCH05 bacterial suspension was sprayed onto the leaves and branches of the seedlings using a hand sprayer until they were completely wet (30 mL/plant). After 24 h, *E. amylovora* suspension was sprayed onto the seedlings. A control treatment of 4,000-fold diluted streptomycin and a control treatment of strain WCH05, and sterile water were also used. Three pots of each treatment were sprayed three times. The inoculated pear seedlings were incubated in a greenhouse at 28–30°C and 70% relative humidity. Disease development was observed daily. The number of diseased branches, the length of diseased branches, the ratio of diseased branch length to the length of the inoculated branches, and the disease severity level were recorded. Disease incidence and disease index were calculated, and efficacy was determined.

##### 2.3.3.2 Therapeutic efficacy

In the therapeutic assay, the inoculation order of myxobacteria and the pathogen was reversed from the protective assay. That means the Ea bacterial suspension was sprayed onto the pear seedlings 24 h after the strain WCH05 bacterial suspension was sprayed. The experimental materials, incubation conditions, and efficacy evaluation were the same as above. The disease scale of fire blight was referred to Li et al. (2021).

### 2.4 Real-time quantitative PCR

The abundances of strain WCH05 in pear (*P. betulifolia*) leaf and pear (*P. sinkiangensis*) flower tissues were quantified using RT-qPCR. Specific primers for RT-qPCR were designed targeting the *lepA* gene (Accession number: ON313804) of strain WCH05, which encodes leader peptidase, a GTP binding membrane protein (Stackebrandt et al., 2007). The primers, *lepA*-F (5′-GGTGTTCGACTCCTGGTACG-3′) and *lepA*-R (5′-CTGAAGACACCCAGCTCCTG-3′) were designed using Primer3Plus online primer design software.^1^ The amplicon size was 141 bp. The *lepA* gene fragment was cloned into T-Vector pMD™19 (Beijing Takara Biomedical Technology Co., Ltd., China) to obtain plasmid pMD19-lepA. Real-time fluorescence quantitative PCR was performed using gradient dilutions of plasmid pMD19-lepA as a template to establish a standard curve (y = −3.2512x + 41.696). RT-qPCR was conducted using a Bio-Rad CFX96 Touch Real-Time PCR Detection System (Bio-Rad, Hercules City, CA, United States) and Takara TB Green® Premix DimerEraser^™^ (Takara, China).

### 2.5 Scanning electron microscopy

The spatial distribution of myxobacteria strain WCH05 within pear flower and leaf tissues following inoculation was meticulously examined using scanning electron microscopy (SEM). Thin sections of the plant material were visualized under a SUPRA55 VP SEM microscope (Zeiss).

### 2.6 Secretome of strain WCH05

To understand strain WCH05’s predatory prowess, we focused on its potent extracellular secretions. The strain WCH05 cultures (incubation 3 days) were centrifuged (12,000 rpm, 10 min), harvest the sterile supernatant with a 0.22 μm filter. The aforementioned supernatant was subsequently applied to cultures of Ea for a duration of 48 h, and samples were obtained at 12, 24, and 48 h post co-culturing to determine the viable cell count of Ea. Further probing their impact, we constructed a unique arena in a petri dish: a membrane-divided battlefield allowing extracellular metabolite infiltration but no cell contact. Here, the strain WCH05 and Ea are separated by this barrier.

### 2.7 Assay of strain WCH05 organic extracts

The strain WCH05 was introduced into the VY/2 culture medium, with the addition of 2% macroporous resin XAD-16. The culture was incubated at 30°C and 180 rpm for 4 days. The macroporous resin was collected by filtration with gauze, and twice the volume of methanol was added to the extraction. The crude extract was obtained by rotary evaporation. Then dissolved in dimethyl sulfoxide DSMO: H_2_O (v/v 1∶1). After co-culturing of extract with Ea for 24 h, the number of residual living Ea cells was determined by serial dilution plating. DMSO was used as the control.

### 2.8 Assay of strain WCH05 extracellular proteins

The strain WCH05 was cultured in VY/2 broth at 30°C for 4 days in a 180-rpm shaker. Following centrifugation at 12,000 rpm, 10 min the supernatant was harvested. Protein in the spent culture was precipitated with ammonium sulfate at various saturations (Li et al., 2019b). Dissolved in PBS buffer (NaCl 8.0 g/L, KCl 0.2 g/L, Na_2_HPO_4_ 1.44 g/L, KH_2_PO_4_ 0.24 g/L, pH 7.2) and cleansed of residual salts via dialysis. Fresh cultured Ea cells (OD_600_ 1.0) were then challenged with this protein arsenal. After 24 h of incubation at 37°C, the battlefield was surveyed. To virtualize the predatory results, transmission electron microscopy (TEM, Hitachi HT7800) was carried out with different scenarios.

### 2.9 Polysaccharide lyase and lipase activity assay

The polysaccharide lyase activity of the extracellular protein was evaluated using pustulan, chitin, xylan, carboxymethyl cellulose, laminarin, yeast glucan, and *β*-1,3-glucan as substrates. Enzyme activity was quantified using the 3,5-dinitrosalicylic acid (DNS) method with a commercially available DNS assay kit (Beijing Solarbio Science Technology Co., Ltd., China) following the manufacturer’s instructions. Lipase activity was determined using p-nitrophenyl palmitate as a substrate, as described by Zheng et al. (2011). A standard curve was generated using a series of diluted p-nitrophenol solutions. Inactivated crude enzyme solution served as a negative control. Each experiment was performed in triplicate.

### 2.10 Statistical analysis

Statistical analysis was performed using a standard analysis of variance (ANOVA) followed by Duncan’s multiple comparison test to identify significant differences among treatments. A significance level of *p* < 0.05 was considered statistically significant. To assess the variability within the data, standard deviations were calculated for all mean values. The ANOVA analysis was conducted using SPSS Statistics 19.0 software.

## 3 Results

### 3.1 *Myxococcus fulvus* WCH05 exhibits active predation against six plant pathogenic bacteria

**Figure 1:**
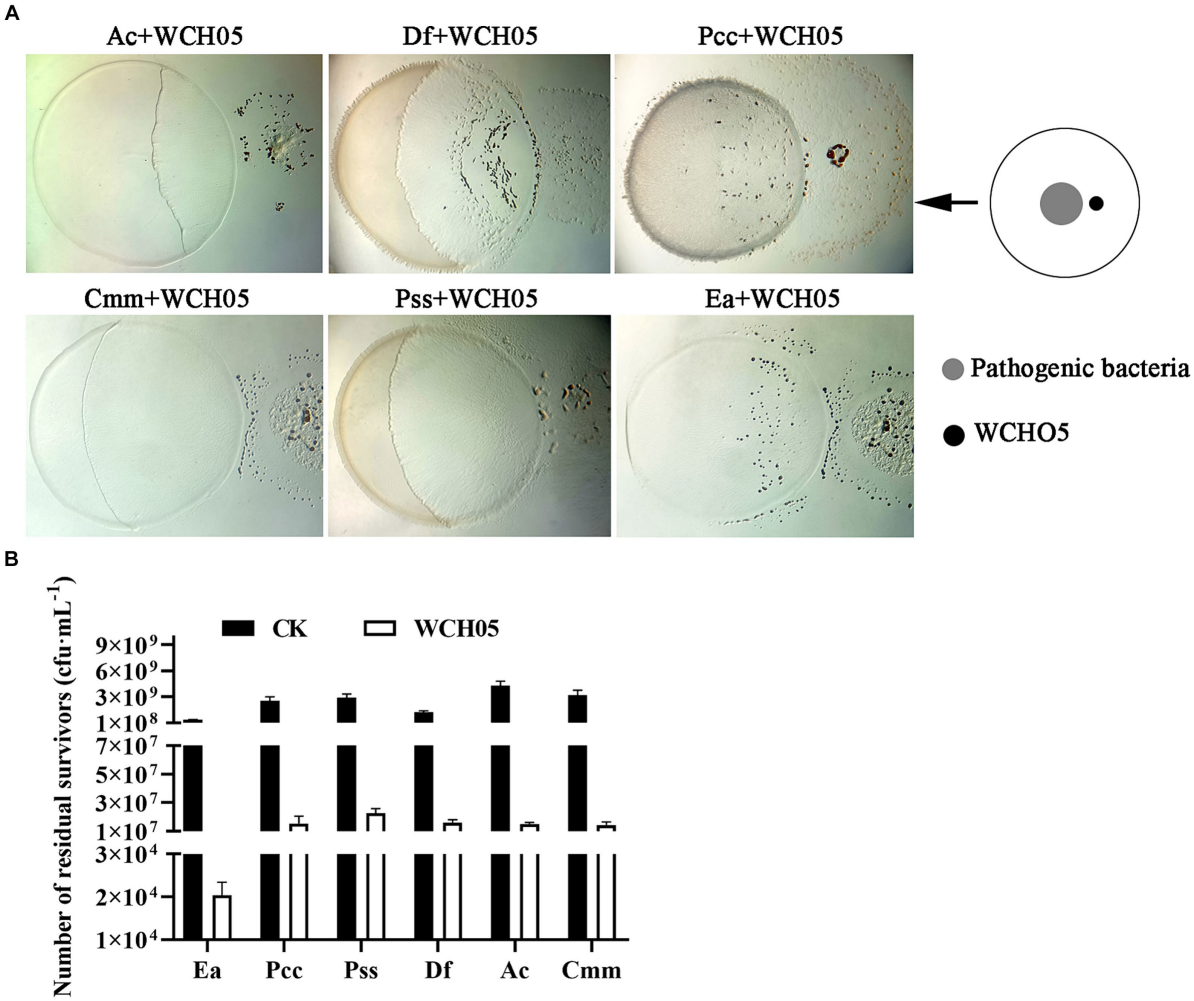
Predatory activity of *Myxococcus fulvus* WCH05 against diverse plant pathogenic bacteria. **(A)** Predation assay setup. Top-down schematic visualization illustrating a 50 μL inoculum of plant pathogenic bacteria placed on TPM plates, followed by a 3 μL spot of strain WCH05 suspension positioned 2 mm away. **(B)** Quantification of predation efficiency. Viable cell counts of various plant pathogenic bacteria after exposure to strain WCH05 on TPM plates.

In this study, we investigated the ability of *Myxococcus fulvus* WCH05 to prey on six plant pathogenic bacteria. We found that strain WCH05 exhibited strong chemotaxis towards all six pathogens, migrating towards and ultimately covering their bacterial lawns (Figure 1A). Co-culture for 5 days resulted in significant reductions in viable cell counts for all pathogens. Ea, for instance, its viable cells declined 5 × 10^3^ times, while the remaining pathogens experienced reductions from 10^9^ cfu·mL^−1^ to 10^7^ cfu·mL^−1^ (Figure 1B). These findings demonstrate strain WCH05’s broad predatory range ability against plant pathogenic bacteria.

**Figure 2:**
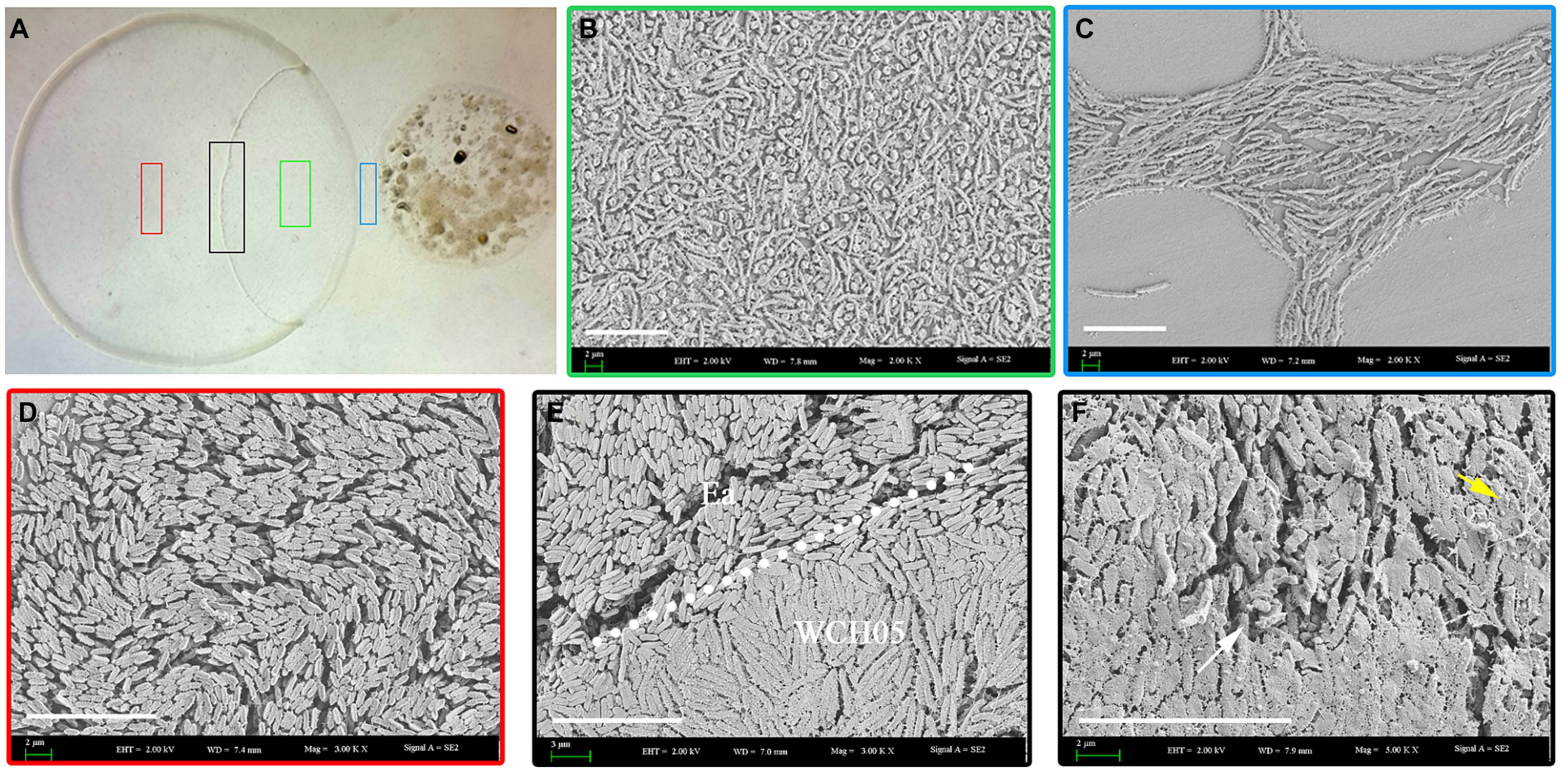
Predation of Ea by *Myxococcus fulvus* WCH05 revealed by scanning electron microscopy (SEM). **(A)** SEM image of strain WCH05 predation on Ea bacteria on a TPM plate. Samples were collected from areas marked by green, blue, black and red boxes in subsequent panels. **(B)** Magnified view of the green box in panel **A**, showing strain WCH05 transformed into numerous myxospores after predation. **(C)** Blue box magnification from panel A reveals strain WCH05 exhibiting tropism towards Ea cells. **(D)** Normal Ea cells with intact morphology, densely distributed on the TPM plate. **(E)** Predation interface where strain WCH05, Ea cells, and intact Ea coexist. The dashed white line delineates the boundary between Ea cells and strain WCH05 cells. **(F)** Close-up view of contact between strain WCH05 and Ea. Ea cells exhibit disrupted morphology and fragmentation. Yellow arrows highlight strain WCH05 secreting a reticulate extracellular substance entangling Ea cells. Scale bars represent 10 μm.

Scanning electron microscopy revealed the intricacies of strain WCH05’s predation on Ea cells. Upon sensing Ea-derived signals, strain WCH05 cells aggregated and moved in a coordinated manner towards Ea colonies (Figure 2C). They subsequently penetrated the Ea clusters (Figures 2E,F), causing morphological disruption and cell shrinkage/rupture (Figure 2F). Notably, damaged Ea cells were enveloped in a network of filamentous material (Figure 2F), likely representing strain WCH05’s extracellular metabolites involved in Ea cell lysis. These results solidify strain WCH05’s potential as a promising candidate for biocontrol development against diverse plant pathogens.

### 3.2 Efficacy of biological control on fire blight

We conducted greenhouse trials to assess the protective and therapeutic efficacy of strain WCH05 against fire blight. The results showed that strain WCH05 was able to significantly reduce the incidence and severity of Ea-induced fire blight.

**Figure 3:**
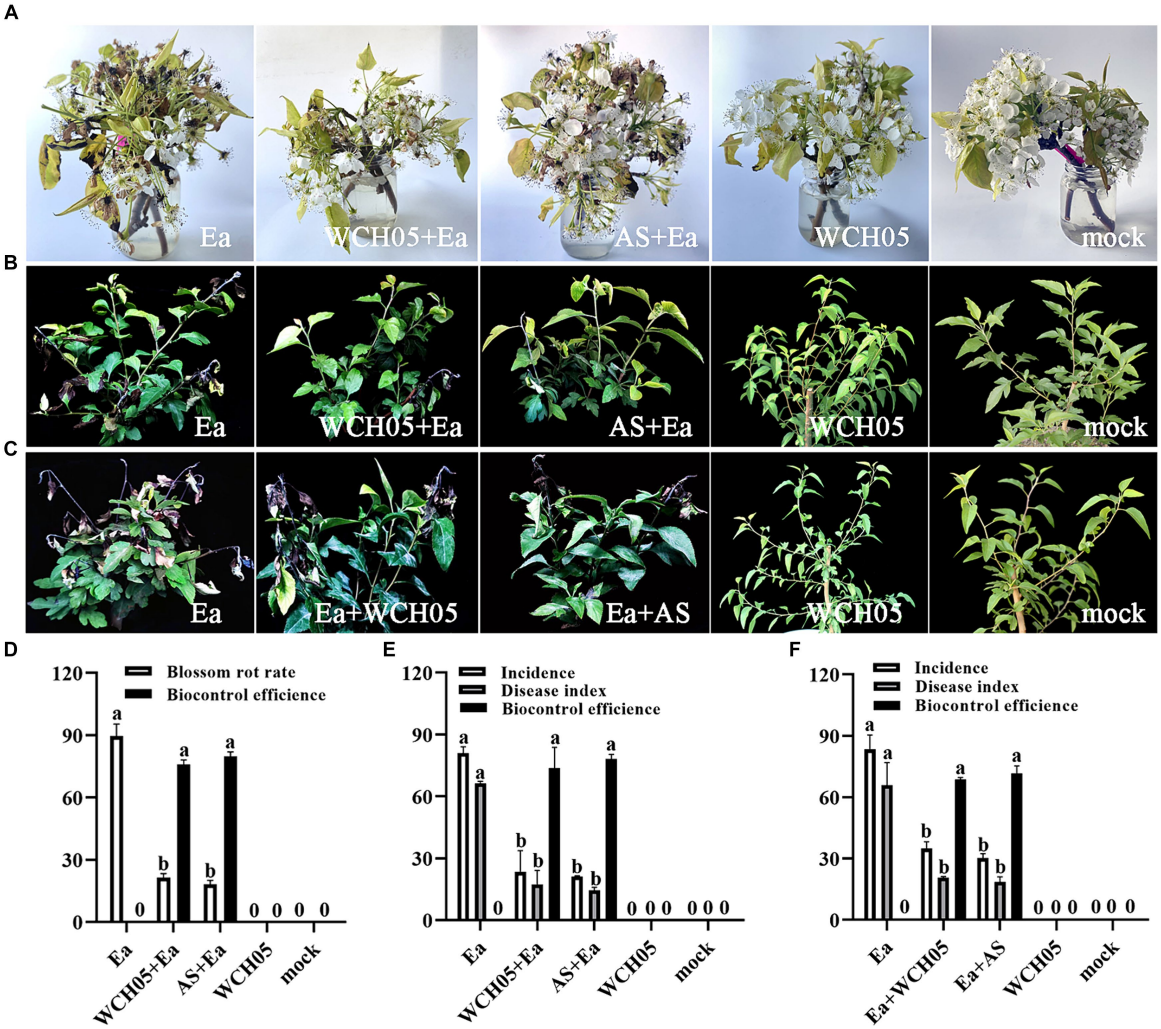
Bioassay of *Myxococcus fulvus* WCH05 for controlling pear fire blight. **(A)** *Ex vivo* pear inflorescence assay for strain WCH05 efficacy against pear fire blight. Protective **(B)** and therapeutic **(C)** assays were conducted to evaluate the efficacy of strain WCH05 against fire blight on pear seedlings. **(D)** Biocontrol efficacy of strain WCH05 against pear blossom rot. **(E)** Protective control effect of the strain WCH05 to fire blight of *Pyrus betulifolia*. **(F)** Therapeutic control effect of strain WCH05 to fire blight of *P. betulifolia.* Mock: Control with sterile water. Ea: Inoculated only with *Erwinia amylovora* (Ea). WCH05, Inoculated only with strain WCH05. WCH05 + Ea, Pre-treated with strain WCH05 24 h before Ea inoculation. AS+Ea, Pre-treated with streptomycin 24 h before Ea inoculation. Ea + WCH05, Inoculated with Ea, then treated with strain WCH05 24 h later. Ea + AS, Inoculated with Ea, then treated with streptomycin 24 h later.

Observations showed that spraying with the viscous bacterium treatment could reduce the flower rot rate. The effective rate on the 5th day was 76.02%, which was close to the effective rate of agricultural streptomycin (79.79%) (*p* = 0.66) (Figures 3A–D). To further study the effect of the viscous bacterium strain WCH05, we used greenhouse potted pear seedlings as the inoculation material and measured the protective and therapeutic effects of strain WCH05. The results of the protective experiment showed that pre-spraying strain WCH05 on pear seedlings could significantly reduce the incidence and disease index of pear seedlings. The protective effect on the 14th day was 73.68%, which was slightly lower than the efficacy of agricultural streptomycin (78.22%) (*p* = 0.96) (Figures 3B,E). The results of the therapeutic experiment showed that spraying strain WCH05 bacterial solution had a significant therapeutic effect on the branch wilt of pear seedlings, but the effective rate decreased. The therapeutic effect on the 14th day was 68.66%, which was slightly lower than the efficacy of agricultural streptomycin (71.57%) (*p* = 0.31) (Figures 3C,F).

**Figure 4:**
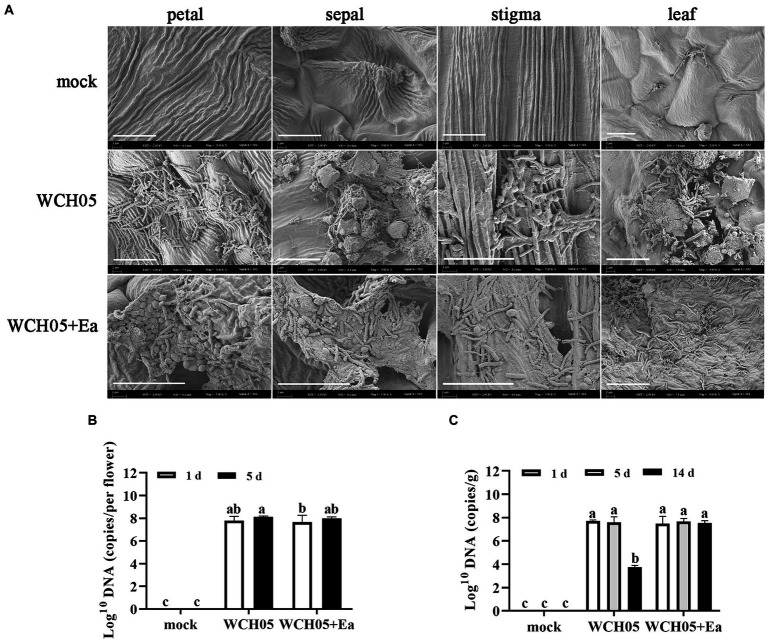
Colonization and quantification of *Myxococcus fulvus* WCH05 on pear inflorescence and leaves. **(A)** Scanning electron microscopy (SEM) images of strain WCH05 colonization on pear flower petals, sepals, stigma, and leaf surfaces. Images were taken 5 days after strain WCH05 inoculation on pear inflorescence and 14 days after strain WCH05 inoculation on pear leaves. **(B)** Quantitative analysis of strain WCH05 DNA copy number on pear inflorescence surfaces 1 and 5 days after strain WCH05 inoculation. **(C)** Quantitative analysis of strain WCH05 DNA copy number on pear leaf surfaces 1, 5, and 14 days after strain WCH05 inoculation. Mock, Control with sterile water; WCH05, Inoculated only with strain WCH05; WCH05 + Ea, Pre-treated with strain WCH05 24 h before Ea inoculation. Scale bars represent 10 μm. Error bars represent standard errors of the mean (±SD) from three independent replicates. Statistical comparisons were performed using Duncan’s multiple range test (*p* < 0.05). Groups marked with the same letter do not differ significantly, while those with different letters show statistically significant differences.

SEM observations showed that strain WCH05 was able to colonize pear leaves and flower clusters. The cells were able to form a dense network on the leaf surface and produce extracellular metabolites that could inhibit the growth of pathogenic bacteria (Figure 4A).

Further quantitative detection found that after strain WCH05 was inoculated into pear flower clusters and pear leaves for up to 5 days, the biomass of strain WCH05 in each treatment did not significantly decrease compared with the first day after inoculation (Figures 4B,C). However, on the 14th day after inoculation on pear leaves, the biomass of strain WCH05 in the treatment group that only inoculated strain WCH05 decreased significantly (*p* < 0.05), while the biomass of strain WCH05 in the WCH05 + Ea treatment group did not change significantly (Figure 4C).

### 3.3 *Myxococcus fulvus* WCH05 predation on Ea relies on direct physical contact

**Figure 5:**
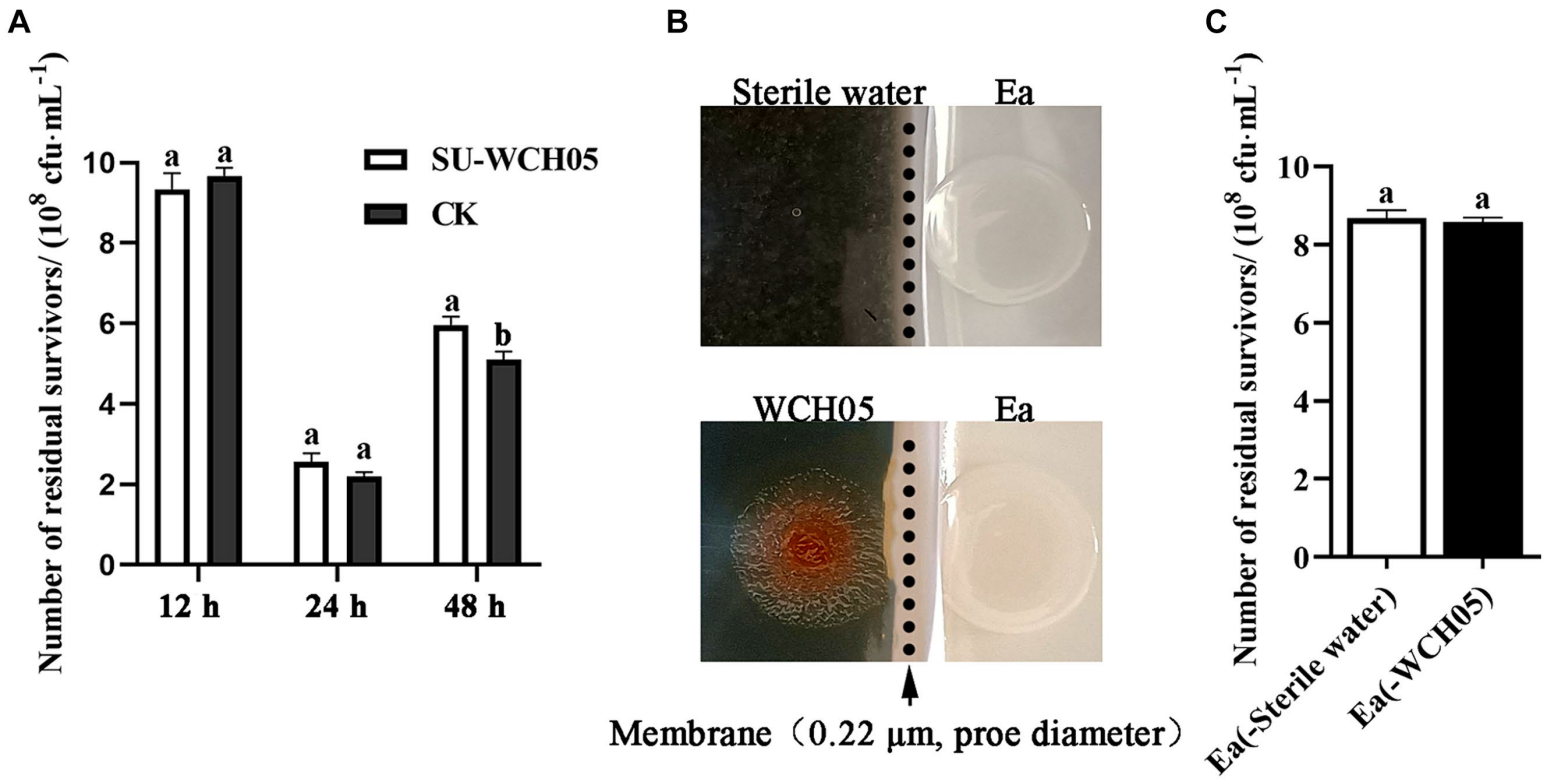
Survival of *Erwinia amylovora* (Ea) under different exposures to *Myxococcus fulvus* WCH05. **(A)** Viable cell counts of Ea after treatment with sterile supernatant of strain WCH05 (SU-WCH05). **(B)** Schematic illustration of strain WCH05 separation from VY/2 medium using a 0.22 μm pore diameter membrane (indicated by the arrow and dotted line). **(C)** Viable cell counts of Ea isolated from the culture were obtained after strain WCH05 separation. Error bars represent standard errors of the mean (±SD) from three independent replicates. Statistical comparisons were performed using Duncan’s multiple range test (*p* < 0.05). Groups marked with the same letter do not differ significantly, while those with different letters show statistically significant differences.

The cell-free fermentation filtrate of strain WCH05 was co-cultured with Ea, and samples were taken at 12 h, 24 h, and 48 h. The number of viable Ea cells was determined by the dilution plating method. The results showed (Figure 5A) that the cell-free fermentation filtrate of strain WCH05 had no inhibitory effect on Ea during co-culture for 12–24 h. There was no significant difference in the number of viable cells remaining compared to the control. At 48 h, the number of viable Ea cells in the cell-free fermentation filtrate was even significantly higher than that in the control (*p* < 0.05) (Figure 5A). This lack of inhibition was mirrored in a separate bacterial membrane isolation experiment, where Ea exposed only to strain WCH05’s secreted metabolites displayed no reduction in viability (Figures 5B,C). Taken together, these results underscore the crucial role of direct bacterial contact in maximizing strain WCH05’s predatory activity against Ea.

### 3.4 Activity of extracellular proteins of strain WCH05

**Figure 6:**
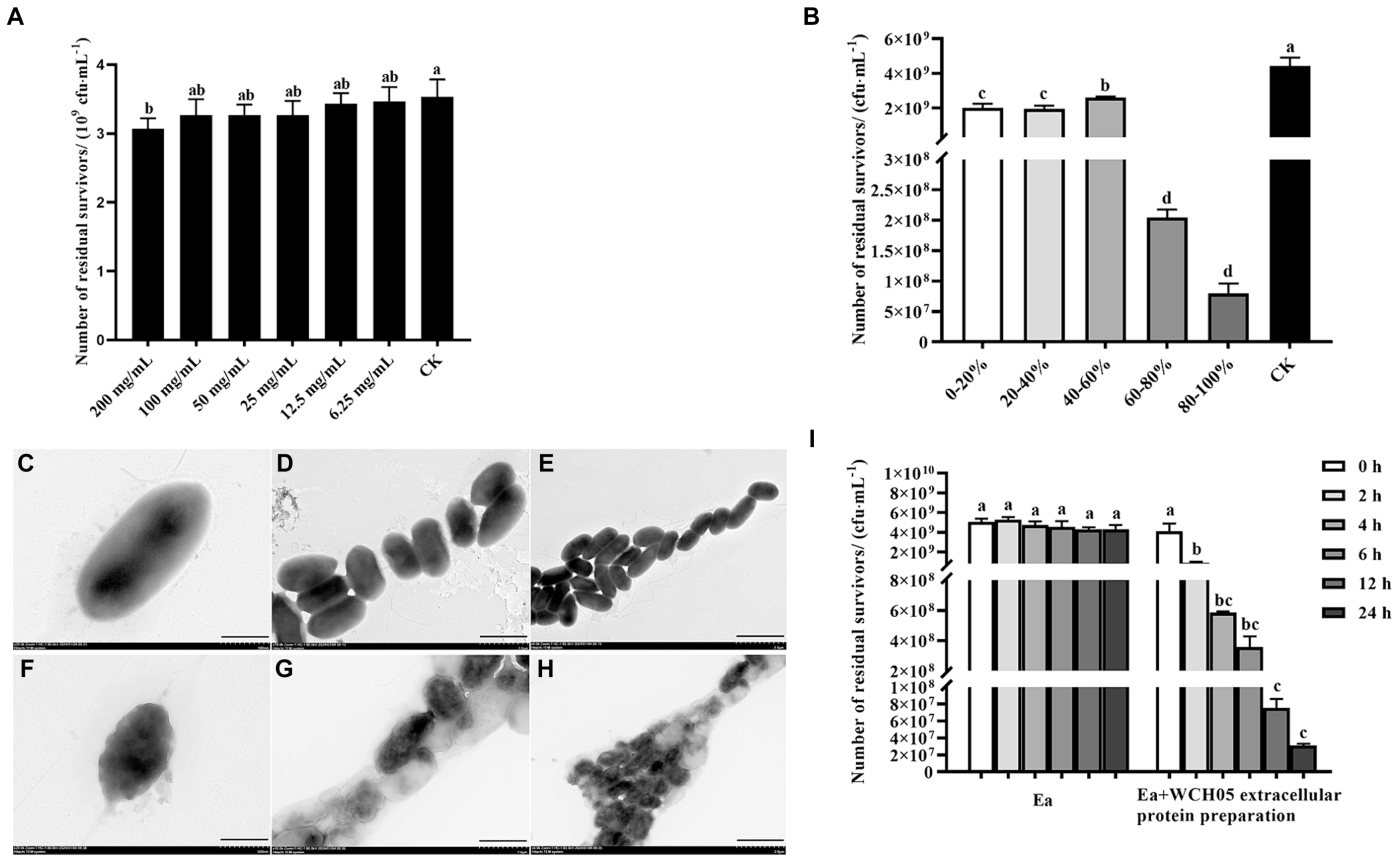
Unveiling the multifaceted attack of *Myxococcus fulvus* WCH05 secretions on Ea. **(A)** Dose-Dependent inhibition of Ea activity by strain WCH05 secondary metabolites. **(B)** Targeting Ea with strain WCH05 extracellular proteins: saturation precipitation strategy. **(C–E)** Transmission electron microscopy of Ea cells exposed to PBS control buffer. **(F–H)** Transmission electron microscopy of Ea cells treated with strain WCH05 extracellular proteins. **(I)** Time-Dependent effect of Ea extracellular proteins on strain WCH05 cell viability. Scale bars: 500 nm for panels C and F, 1 μm for panels **(D,G)**, 2 μm for panels **(E,H)**. Error bars represent standard errors of the mean (±SD) from three independent replicates. Statistical comparisons were performed using Duncan’s multiple range test (*p* < 0.05). Groups marked with the same letter do not differ significantly, while those with different letters show statistically significant differences.

Secondary metabolites extracted from the supernatant fermentation broth of strain WCH05 using a large-pore resin were diluted to different concentrations and co-cultured with Ea cells for 24 h to determine their inhibitory activity. The results revealed that the crude extract exhibited significant inhibitory activity against strain Ea only at a concentration of 200 mg/mL compared to the control. However, the viable cell counts of Ea decreased only slightly from the control 3.5 × 10^9^ cfu·mL^−1^ to 3.0 × 10^9^ cfu·mL^−1^. Therefore, it is inferred that the secondary metabolites secreted by strain WCH05 cells did not play a major role (Figure 6A).

Furthermore, this study extracted the extracellular crude protein of strain WCH05 and determined its inhibitory activity against Ea. The results showed that the protein component treated with saturated ammonium sulfate at 60–80% led to a 1-log decrease in the viable cell number of Ea, while the protein component obtained by saturated ammonium sulfate at 80–100% led to a 2-log decrease in the viable cell number of Ea. The antibacterial activity of protein fractions precipitated by 20–40% and 40–60% saturated ammonium sulfate exhibited relatively low potency (Figure 6B).

Transmission electron microscopy observations revealed that the cell structure of Ea was loose and irregular, the cell contents were overflowing, and the integrity was destroyed (Figures 6C–H). These results suggest that effective proteins may exist in the components of saturated ammonium sulfate at 60–100%.

Further detection of the changes in the number of viable Ea cells before and after extracellular protein treatment was carried out using the plate gradient dilution method. The results showed that the number of viable cells significantly decreased with the prolongation of time after treatment with the extracellular protein of strain WCH05 (Figure 6I).

### 3.5 Activities of various enzymes

**Figure 7:**
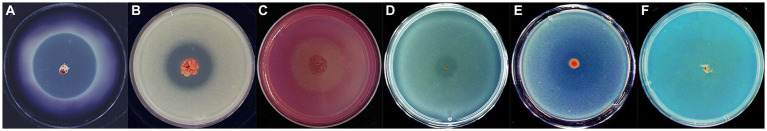
Assay of *Myxococcus fulvus* WCH05 degradation of various polymers. Each panel **(A–F)** shows a CTT plate containing 0.5% of a specific polymer: **(A)** starch, **(B)** skimmed milk, **(C)** sodium carboxymethyl cellulose, **(D)** tributyrin, **(E)** *β*-glucan, and **(F)** colloidal chitin. An exponential culture of strain WCH05 was spotted on each plate and incubated at 30°C for 5 days. The images shown are representative of triplicate experiments.

We investigated the lytic activity of strain WCH05 towards various substrates. The results demonstrated its ability to degrade starch, skimmed milk, sodium carboxymethyl cellulose, tributyrin, and glucan (Figures 7A,E). Notably, chitin proved resistant to degradation (Figure 7F).

To further characterize the substrate spectrum of its lytic extracellular proteins, we conducted additional analyses. The proteins readily hydrolyzed xylan with *β*-1,4-xylopyranosyl bonds, carboxymethyl cellulose with *β*-1,4 glycosidic bonds, laminarin with *β*-1,3–1,6 glycosidic bonds, Yeast glucan with *β*-1,3–1,6 glycosidic bonds, *β*-1,3-glucan with *β*-1,3 glycosidic bonds, and the lipase substrate p-nitrophenyl palmitate (Table 1). Conversely, they exhibited no activity towards pustulan with *β*-1,6-glycosidic bonds or chitin. These findings suggest that the extracellular proteins of strain WCH05 likely possess lipase, cellulase, and glycoside hydrolase activity specifically targeting *β*-1,3-glycosidic bonds.

**Table 1:** Substrate specificity of extracellular crude enzymes of strain WCH05.

Substrate	Bond types	Enzyme activity (U·mL^−1^)
Pustulan	*β*-1,6-(Glucose)	0
Chitin	*β*-1, 4-*N*-Acetylaminoglycoside bond	0
p-Nitrophenyl palmitate	Ester linkage	82.24 ± 2.87
Xylan	*β*-1,4-(Xylopyranosyl)	21.02 ± 0.41
Carboxymethyl cellulose	*β*-1,4-(Glucose)	8.97 ± 0.39
Laminarin	*β*-1,3-*β*-1,6-(Glucose)	6.68 ± 0.61
Yeast glucan	*β*-1,3-*β*-1,6-(Glucose)	6.17 ± 0.55
*β*-1,3-glucan	*β*-1,3-(Glucose)	6.14 ± 0.10

## 4 Discussion

The invasion of fire blight poses a severe threat to China’s fruit industry, particularly imposing significant risks on the pear industry in Xinjiang. In recent years, the role of beneficial microorganisms as biocontrol agents in the biological control of fire blight has received considerable attention and has shown promising results. While numerous microorganisms have been employed for the biological control of fire blight, research, and application of myxobacteria in this context remain largely unexplored. Extensive studies in recent years have highlighted the significant potential of myxobacteria in the biological control of plant diseases. In the field of combating plant pathogenic fungi, *Corallococcus* (Zhou et al., 2019; Li et al., 2019a; Xia et al., 2023), *Myxococcus* (Dong et al., 2021; Wu et al., 2021), *Nannocystis exedens* (Taylor and Draughon, 2001), *Cochliobolus miyabeanus* (Homma, 1984), and other predatory myxobacteria (Taylor and Draughon, 2001; Meliah et al., 2020), have demonstrated effective biocontrol against various plant pathogenic fungi. Notably, myxobacteria exhibit superior predation and antagonistic effects against bacteria, showcasing broad prospects in the biological control of bacterial plant diseases. For instance, *Myxococcus* sp. BS effectively inhibits the infection of carrot soft rot *Pectobacterium carotovorum* subsp*. carotovorum* on calla lily (Li et al., 2018). *M. xanthus* R31 exhibits significant biocontrol efficacy against tomato bacterial wilt (Dong et al., 2021). However, there are no report on fire blight using the predatory myxobacteria. To our knowledge, this is the first report of fire blight biocontrol using predatory myxobacteria.

This study, through detached flower cluster and potted Chinese pear seedling inoculation experiments, provides the first evidence of the potential application of myxobacteria in the biological control of fire blight. The findings not only offer new microbial resources for the biological control of fire blight but also lay the foundation for further research and development of myxobacterial biocontrol agents in the context of fire blight.

To date, the isolation and application of antagonistic bacteria for the biological control of plant pathogens remain a highly active research area. Researchers have identified numerous antagonistic strains with inhibitory effects against plant pathogens, including *Bacillus*, *Pseudomonas*, *Streptomyces*, *Lysobacter*, and *Trichoderma*, among others (Law et al., 2017; Fira et al., 2018; Sallam et al., 2019; Lin et al., 2021). The biocontrol mechanisms of these strains primarily involve the production of various antibiotic-like substances, toxins, bacteriocins, and proteinaceous antimicrobial substances during their growth metabolism, leading to the inhibition or elimination of plant pathogens. Myxobacteria, through their unique wolf-pack collective behavior and gliding motion, actively prey on bacteria, fungi, and yeast microorganisms (Mohr et al., 2016). However, effective predation by myxobacteria appears to involve direct cell-to-cell contact. Previous observations have demonstrated that myxobacterial strains, such as *Nannocystis exedens*, exert inhibitory effects on *Aspergillus flavus* and *A. parasiticus* by direct contact via the bacterial membrane, lysing the spores, hyphae, and nuclei of the predatory fungi (Taylor and Draughon, 2001). Similarly, in this study, myxobacterial strain WCH05 exhibited efficient predatory capabilities upon contact with the *E. amylovora* on solid agar plates. However, the activity of Ea cells remained unaffected in co-culture experiments using sterile fermentation filtrate and membrane-separated co-culture experiments, reaffirming that the effective killing of prey cells by myxobacteria depends on direct cell-to-cell contact.

Currently, the predation mechanisms of myxobacteria primarily involve secondary metabolites, lytic enzymes, outer membrane vesicles (OMVs), and others (Berleman and Kirby, 2009; Evans et al., 2012; Mohr et al., 2016). Among these, the secondary metabolites produced by myxobacteria are considered small-molecule weapons capable of penetrating prey cells, halting their metabolism, or causing cell death (Xiao et al., 2011). For instance, the antibiotic TA produced by *M. xanthus* DK1622 exhibits potent antibacterial activity against *E. coli* MG1655 but lacks inhibitory activity against the Gram-positive bacterium *Micrococcus luteus*, suggesting a selective antibacterial effect. Furthermore, the efficacy of TA is influenced by the physiological state of prey cells, with reduced activity observed in metabolically inactive cells (Goldman et al., 2006; Xiao et al., 2011). Corallopyronin produced by *Corallococcus coralloides* exerts antibacterial effects by inhibiting bacterial RNA synthesis, predominantly inhibiting Gram-positive bacteria while having no impact on Gram-negative bacteria, yeast, and molds (Irschik et al., 1985). Recent advancements have elucidated the predatory mechanisms of myxobacteria, particularly highlighting the role of the type III and IV secretion systems. Seef et al. (2021) demonstrated that the integration of A-motility with contact-dependent killing serves as the primary predatory strategy for efficient invasion and consumption of prey colonies on surfaces. This process is mediated at the molecular level by a novel type IV filament-like apparatus (Kil), which facilitates both the immobilization of the predator and the plasmolysis of prey cells. Further investigations by Thiery et al. (2022) into the molecular mechanisms underlying contact-dependent killing in *M. xanthus* focused on the analysis of four protein secretion systems. Their research on the predation dynamics of mutant strains over various timescales revealed that a Tad-like and a type 3-like secretion system (Tad and T3SS) play pivotal roles in prey interaction. Specifically, the Tad-like system is crucial for inducing prey cell death, whereas the T3SS, despite lacking a needle structure, is responsible for initiating prey cell lysis.

In this study, we found that the sterile fermentation filtrate of strain WCH05 showed no inhibitory activity against Ea, although it cannot be ruled out that this may be due to a low content of secondary metabolites. Therefore, we further extracted secondary metabolites from the sterile fermentation filtrate using macroporous resin, The results revealed that secondary metabolites did not play a major role in the antibacterial activity. Consequently, we speculate that the secondary metabolites produced by strain WCH05 may not play a major role in the biological control of fire blight. Similarly, Dong et al. (2021) found that secondary metabolites secreted by *M. xanthus* R31 showed no inhibitory activity against *R. solanacearum*.

Myxobacteria can produce various enzymes, including proteases, amylases, cellulases, lipases, chitinases, xylanases, and others (Munoz-Dorado et al., 2016), forming the material basis for their predation. Previous studies suggested that myxobacteria secrete proteases or peptidases, lysozymes, and other lytic enzymes that may participate in their predation, but direct evidence has been lacking thus far (Ensign and Wolfe, 1966; Berleman and Kirby, 2009). Arend et al. (2021) demonstrated that *M. xanthus* employs different antibacterial mechanisms for predating Gram-positive or Gram-negative bacteria. For Gram-positive bacteria, *M. xanthus* secretes proteins that degrade peptidoglycan layers, leading to cell lysis, while outer membrane vesicles (OMVs) play a crucial role against Gram-negative bacteria. Transcriptome analysis of myxobacteria predation on *Escherichia coli* revealed the activation of thousands of genes, suggesting that the bacterial cell wall and proteins are primary targets of myxobacterial attack (Livingstone et al., 2018). In the predation process of *Corallococcus* sp. EGB on fungi, the strain disrupts the fungal cell wall by secreting GluM and CcCti1 (Li et al., 2019a,b). These findings suggest that the enzymes secreted by myxobacteria during predation may be related to the composition of the prey cell wall. In this study, we observed that the extracellular enzymes produced by strain WCH05 exhibited lytic activity against Ea. Thus, we speculate that some of the extracellular enzymes, especially peptidases, lipases, glycoside hydrolases, etc., produced by strain WCH05 play a crucial role in its predation on Ea and contribute to the biological control of fire blight. However, the types of lytic enzymes secreted by myxobacteria during predation and their modes of action on prey cells require further investigation through transcriptomic and proteomic analyses. Future studies on the interactions between myxobacteria, prey, and plants will help elucidate the predation mechanisms of myxobacteria.

Myxobacteria are widely distributed in soil (Zhou et al., 2014), exhibiting strong adaptability to soil environments, making them more prone to colonization in soil. This is likely one of the reasons why research and application of myxobacteria in biological control have primarily focused on soil-borne diseases. In contrast to soil environments, the leaf surface represents a more challenging survival environment for microorganisms, with limited available nutrients and significant fluctuations in temperature, humidity, and UV radiation, all of which can profoundly impact microbial survival (Vorholt, 2012). As indigenous soil bacteria, whether myxobacteria can establish residency on the phyllosphere of plants remains unexplored.

SEM observation revealed that after strain WCH05 was inoculated onto pear leaf and inflorescence surfaces, it appeared to be able to adhere to the leaf and inflorescence surfaces via a large number of extracellular metabolites secreted by the cells (Figure 4A). Further detection by real-time quantitative PCR showed that strain WCH05 was able to maintain stable biomass on both pear inflorescence and *P. calleryana* leaf surfaces for the first 5 days after inoculation. This may be due to the broad-spectrum predatory activity of myxobacteria, simple nutrient requirements, and the fact that the epiphytic microorganisms and nutrients on the surface of plant tissues can meet the nutrient requirements of myxobacteria in a short period (Figures 4B,C). By the 14th day, with the gradual depletion of nutrients, coupled with the autolysis characteristics of myxobacteria growth, the biomass of strain WCH05 on the Ea-uninoculated *P.calleryana* leaf surface decreased significantly. However, on the Ea-inoculated *P. calleryana* leaf surface, strain WCH05 was able to continuously obtain nutrients through predation, thus maintaining the stability of the bacterial community (Figure 4C).

Similarly, Eisner et al. (2023) found that the wheat pathogenic fungus *Zymoseptoria tritici* on wheat straw could promote the growth of *M. xanthus*, suggesting potential reasons such as direct predation, release of carbon sources by *Z. tritici*, and alteration of environmental conditions favoring *M. xanthus* growth. However, the specific mechanisms underlying this growth promotion need further investigation for confirmation.

Although this study confirmed the significant biocontrol effect of myxobacteria against fire blight under greenhouse conditions and their ability to colonize the surfaces of pear flowers and leaves, their resistance, colonization efficiency, and control efficacy in the natural field environment need further confirmation in subsequent work. Additionally, some bottleneck issues limit the practical application of myxobacteria in the biological control of fire blight. For instance, the inherent autolytic characteristics of myxobacteria directly constrain the large-scale preparation and shelf life of myxobacterial agents. The tendency of myxobacteria to aggregate during growth in liquid culture severely hampers spray application. Therefore, addressing how to enhance cell dispersion during the growth process, establishing and optimizing myxobacterial fermentation processes, and overcoming these challenges require in-depth investigation in future studies.

## 5 Conclusion

Fire blight, a devastating bacterial disease of pome fruits, demands innovative control strategies. This study introduces *Myxococcus fulvus* WCH05, a potent biocontrol agent with a multifaceted attack against the fire blight pathogen, *E. amylovora*. The strain WCH05 exhibits broad-spectrum activity against plant pathogens through direct predation via a direct contact method. Additionally, extracellular enzyme proteins secreted by strain WCH05, especially certain peptidases, lipases, and glycoside hydrolases, play significant roles in the predation process. *In vivo* assays demonstrated strain WCH05’s efficacy in protecting pear blossoms and young seedlings, rivaling the antibiotic oxytetracycline. This eco-friendly approach offers promising potential for sustainable fire blight management.

However, challenges like large-scale production and application remain. Future research will focus on characterizing strain WCH05’s enzymes and optimizing application methods to translate this exciting biocontrol potential into practical solutions. The strain WCH05 represents a significant step towards sustainable fire blight control and highlights the vast potential of microbial resources for safeguarding crop health and promoting environmentally friendly agriculture.

## Data availability statement

The original contributions presented in the study are included in the article/supplementary material, further inquiries can be directed to the corresponding authors.

## Author contributions

JH: Writing – original draft, Writing – review & editing, Conceptualization, Data curation, Formal analysis, Funding acquisition, Investigation, Project administration, Resources, Supervision. ZD: Data curation, Writing – original draft, Formal analysis, Investigation. WJ: Data curation, Writing – original draft, Formal analysis, Investigation. WL: Data curation, Formal analysis, Writing – original draft. ML: Conceptualization, Funding acquisition, Resources, Supervision, Writing – review & editing. BF: Data curation, Formal analysis, Writing – original draft, Writing – review & editing.

## Footnotes

^1^ https://www.primer3plus.com/index.html
